# Accurate prognostic awareness is associated with increased emotional distress in Latino patients with advanced cancer

**DOI:** 10.1017/S1478951525000252

**Published:** 2025-04-22

**Authors:** Pedro E. Pérez-Cruz, María Jesús San Martín, Josefa Palacios, Laura Tupper-Satt, Marcela González-Otaíza, Paula Repetto

**Affiliations:** 1 Sección de Medicina Paliativa, Facultad de Medicina, Pontificia Universidad Católica de Chile, Santiago, Chile; 2 Centro para el Control y la Prevención del Cáncer (CECAN), Pontificia Universidad Católica de Chile, Santiago, Chile; 3 Escuela de Psicología, Facultad de Educación, Psicología y Familia, Universidad Finis Terrae, Santiago, Chile; 4 Programa de Medicina Paliativa y Cuidados Continuos, Facultad de Medicina, Pontificia Universidad Católica de Chile, Santiago, Chile; 5 Unidad de Cuidados Paliativos, Complejo Asistencial Dr. Sótero del Río, Servicio de Salud Metropolitano Sur Oriente, Puente Alto, Chile; 6 Escuela de Psicología, Pontificia Universidad Católica de Chile, Santiago, Chile

**Keywords:** Awareness, cancer, emotional distress, Latino, palliative care, prognosis

## Abstract

**Objectives:**

To describe the frequency of prognostic awareness (PA) in a population of advanced cancer patients in a Latino community and to explore the relationship between accurate PA with emotional distress and other covariates.

**Methods:**

In this cross-sectional study performed in Puente Alto, Chile, advanced cancer patients in palliative care completed a survey that included a single question to assess PA (Do you believe your cancer is curable? yes/no). Patients reporting that their cancer was not curable were considered as having accurate PA. Demographics, emotional distress, quality of life, and patient perception of treatment goals were also assessed. Analyses to explore associations between PA and patient variables were adjusted.

**Results:**

A total of 201 patients were included in the analysis. Mean age was 65, 50% female. One hundred and three patients (51%) reported an accurate PA. In the univariate analysis, accurate PA was associated with not having a partner (*p* = 0.012), increased emotional distress (*p* = 0.013), depression (*p* = 0.003), and were less likely to report that the goal of the treatment was to get rid of the cancer (*p* < 0.001). In the multivariate analysis, patients with accurate PA had higher emotional distress or depression, were less likely to have a partner, and to report that the goal of the treatment was to get rid of the cancer.

**Significance of results:**

Half of a population of Latino advanced cancer patients reported an accurate PA. Accurate PA was associated with increased emotional distress, which is similar to what has been reported in other countries. Weaknesses in prognostic disclosure by clinicians, local cultural factors, or higher motivation to seek prognostic information among distressed cancer patients could explain this association. Strategies to emotionally support patients when discussing prognostic information should be implemented.

## Introduction

Palliative care (PC) has been proposed as an approach to focus on improving the quality of life of patients with cancer through the prevention and relief of suffering (IAHPC Pallipedia). Several publications have provided evidence that early inclusion of PC in cancer patient care positively benefits patients and their families, including improved symptom control, better quality of life, satisfaction with care, and increased prognostic awareness (PA), among others (Hui and Bruera 2016).

PA has been described as the extent to which patients are aware of their terminal prognosis or shortened life expectancy (Applebaum et al. 2014). It has been proposed that PC specifically helps patients cultivate their PA throughout the disease trajectory, allowing patients to incorporate the prognostic information provided by clinicians at their own pace (Jackson et al. 2013). Global literature suggests that providing patients with adequate prognostic information is crucial for enhancing patient autonomy, helping patients to plan for the remaining time, decreasing the use of health resources during the end-of-life period (Butow et al. 2020). It has been reported that having accurate PA has positive impacts on decision-making at the end-of-life, such as facilitation of treatment planning (Applebaum et al. 2014). Regardless of these positive effects, several studies have shown that at best, only around 50% of people diagnosed with a terminal illness report accurate PA (Chen et al. 2017; Yennurajalingam et al. 2018b).On the other hand, almost half of the population with advanced cancer still have inaccurate PA, a finding reported in different studies across a wide variety of countries (Chen et al. 2017).

Several reports have assessed the relationship between PA and emotional distress, and the findings have been inconsistent. Emotional distress is an unpleasant and multifactorial emotional experience that can move between common feelings of sadness and vulnerability to more disabling problems such as depressive and anxiety disorders and can interfere with patients’ ability to cope with cancer effectively (Holland et al. 2013). Some studies have identified that increased PA is associated with decreased emotional distress, depression, and/or anxiety, supporting the relevance of promoting PA in cancer patients, as it improves patients’ psychological outcomes (Chochinov et al. 2000; George et al. 2020; Innes and Payne 2009; Lichtenthal et al. 2009). Other studies have shown otherwise, with increased PA associated with higher emotional distress, questioning the benefits of promoting patient awareness of their terminal condition (Chen et al. 2023; Ozdemir et al. 2022; Tang et al. 2019). To account for the heterogeneity in the relationship between PA and QOL outcomes, scholars have suggested that culture should be considered as the relationship could vary according to the cultural context (Chen et al. 2017; Ng and Ozdemir 2023; Yennurajalingam et al. 2018b).

To better understand the factors related to PA, several studies have explored its associations with patient demographics and outcomes. Age, gender, educational level, proximity to death, religiosity, and social contact have all been identified as predictors of accurate PA (Vlckova et al. 2020). Decreased functional and cognitive function have also been associated with increased awareness of terminality (Kurita et al. 2018). A higher level of PA has been associated with better quality of life, with the relationship being also inconsistent across studies (Fan et al. 2011; Ng and Ozdemir 2023). Other factors that have been reported to influence patient PA include factors related to coping strategies (e.g. hope, trust in medical professions) (Kavradim et al. 2013), factors related to health status (e.g. cancer type, symptom intensity), and factors associated with caregivers (e.g. relatives PA) (Duberstein et al. 2018; Justo Roll et al. 2009). Another relevant group of factors that have been associated with PA are factors associated with communication (van der Velden et al. 2020; Wattanapisit et al. 2021). For example, strategies and styles of patient–physician communication about prognosis or the amount of information provided have been associated with the frequency of PA (Enzinger et al. 2015; Epstein et al. 2016).

Until now, only one study, assessing patients at a single country have reported the frequency of PA in Latino patients with advanced cancer. This study performed in Cuba, included 91 advanced cancer patients and found that 9% of patients understood that their cancer was not curable (Chen et al. 2017). In this study, the authors did not explore the associations between PA and other variables. Therefore, there is few information regarding how frequently patients report that their disease is not curable and the associations between PA and patient characteristics. Having this information can contribute to identify characteristics that are associated with an accurate understanding of prognosis among Latino patients and to confirm or reject whether what has been found elsewhere can be applied to this culture. Specifically, it seems relevant to explore the association between PA and emotional distress in Latino communities as family caregivers frequently raise their concern about how delivering prognostic information may worsen patients’ emotional well-being. These findings could contribute to suggesting clinicians’ with specific recommendations when disclosing prognostic information for patients in this culture.

The main aims of this study are to describe the frequency of PA in a population of advanced cancer patients in a Latino community, to explore the relationship between accurate PA and emotional distress, and finally, to identify covariates associated with accurate PA in this population.

## Methods

### Study design and participants

This cross-sectional observational study analyses baseline characteristics of advanced cancer patients enrolled in a longitudinal study that aimed to examine patients’ quality of life over time until the patient’s death. Briefly, advanced cancer patients in PC were enrolled at a public hospital in Santiago, Chile, between January 2016 and January 2017 and were followed up every 2 weeks until demise. Inclusion criteria included being 18 years old or older, having a diagnosis of advanced cancer, not presenting delirium as measured by the Memorial Delirium Assessment Scale (MDAS), and a Karnofsky Performance Status (KPS) ≤80. Patients new to PC and patients with previous PC assessments were eligible for participation. After consent, a research nurse trained in PC helped patients complete a baseline questionnaire and then performed the phone surveys to assess patients over time.

### Measures

Patients’ baseline assessments included demographic information, including age, gender, marital status, education, religion, and cancer diagnosis. We used the KPS to assess functionality through an index ranging from 100 (normality and absence of disease) to 0 (death). The KPS is commonly used by health professionals to measure patients’ functional status globally and to predict their survival chances. For each patient, we also estimated the number months since diagnosis of cancer and the number of months since enrolled into PC at the time of the interview.

To assess PA, we asked the following question: “Do you think your cancer is curable?” patients answering “no” were considered to having an accurate PA, and those responding “yes” deemed with inaccurate PA, as all patients had advanced incurable cancer. Although there are different strategies to assess PA, which have been extensively described in the literature (Mathews et al. 2024), we decided to consider a single-item question that has been previously used in different reports, that is simple to understand for advanced cancer patients (Iconomou et al. 2002). This variable was identified as our primary outcome of interest. We also included 3 questions to assess patient perception of the goals of cancer treatments. Specifically, we asked patients whether the goals of the treatments they were receiving were to get rid of the cancer, to live longer, and to improve their quality of life.

To assess emotional distress, we used the Hospital Anxiety and Depression Scale (HADS) in its Spanish validated form (Quintana et al. 2003; Villoria and Lara 2018). This instrument is a self-applied questionnaire with 14 Likert-scale items (Zigmond and Snaith 1983). The 14 items of the questionnaire provide 2 subscales, 1 for anxiety (HADS-A) and 1 for depression (HADS-D). Each subscale has values ranging from 0 to 21, with higher scores reflecting more depressive and anxious symptoms. Although there are different ways to score these scales, values equal or higher than 14 in the HADS total score, equal or higher than 8 in the HADS-A or the HADS-D scales, identify patients with clinically significant emotional distress, anxiety, or depression, respectively. We followed these thresholds to binarily classify patients with or without emotional distress, anxiety, and/or depression.

Patients were asked to report on their health status (good, fair, or bad) and how they felt considering their condition (healthy, relatively sick, or very sick). They were also asked to report on the treatments they have received (surgery, chemotherapy, radiation therapy, or complementary/alternative medicine).

### Statistical considerations

Descriptive statistics were used to summarize collected data. Sample size, mean, and standard deviation (SD) were reported for continuous normally distributed variables. For non-normally distributed variables, median and interquartile range (IQR) were used. For categorical and binary variables, frequencies and percentages were reported. We included patients with all data collected. Univariate analysis was performed, using PA as the primary outcome of interest. We explored the association between each of the variables with PA. *t*-test, chi-square test, or Wilcoxon rank-sum test were used as needed. The main association of interest was the relationship between emotional well-being measured with the total HADS score and PA (main outcome). To assess the relationship between emotional distress and PA, we used logistic regression models using the total HADS score initially, and then we assessed the relationship with the HADS-A and HADS-D subscales. We then included relevant covariates in the final model. All analyses were carried out using a standard software package (Stata, version 12.0; StataCorp).

### Data protection and confidentiality

All procedures of this study were approved by the local Ethics Committee (Comité Ético Científico – Facultad de Medicina, Pontificia Universidad Cat\ólica de Chile, Protocol Number #13-154) and were conducted in accordance with the principles embodied in the Declaration of Helsinki. All participants provided signed informed consent. Health information was protected, and data confidentiality was maintained throughout the study. Only trained personnel in maintaining confidentiality and the primary investigator had access to study records (Perez-Cruz et al. 2017).

## Results

A total of 201 advanced cancer patients were included. Patient demographics are described in Table 1. The mean age of patients was 64 years and 100 (50%) were female. One hundred and three (51%) patients had accurate PA, meaning that they reported to know that their cancer had no cure. Ninety-eight (49%) patients reported that they believed that their cancer could be cured and were deemed to have inaccurate PA.

**Table 1.** Demographic and clinical characteristics of the sample

	Total	Accurate prognostic awareness	Inaccurate prognostic awareness	
	*N* = 201	*N* = 103	*N* = 98	
	*N* (%)	*N* (%)	*N* (%)	*p*-value*
Female sex	100 (50)	53 (51)	47 (48)	0.62
Age (mean [st. deviation (SD)])	64 (14)	65 (14)	62 (14)	0.118^†^
Educational level				0.39
No education	8 (4)	6 (6)	2 (2)	
Middle school or less	149 (74)	75 (73)	74 (76)	
High school or higher	44 (22)	22 (21)	22 (22)	
Marital status				**0.012**
With spouse or partner	126 (63)	56 (54)	70 (71)	
Without spouse or partner (single or widowed)	75 (37)	57 (46)	28 (29)	
Religion				0.051
Catholic	117 (58)	63 (61)	54 (55)	
Evangelical	40 (20)	13 (13)	27 (28)	
Other	19 (9)	12 (12)	7 (7)	
Atheist or agnostic	25 (13)	15 (15)	10 (10)	
Religiosity				0.11
Very or moderately religious	139 (69)	66 (64)	73 (74)	
A little or not religious	62 (31)	37 (36)	25 (26)	
Spirituality (*N* = 199)				0.057
Very or moderately spiritual	148 (74)	70 (69)	78 (80)	
A little or not spiritual	51 (26)	32 (31)	19 (20)	
Cancer type				0.384
Gastrointestinal	80 (40)	45 (44)	35 (36)	
Lung	33 (16)	19 (18)	14 (14)	
Nephrourologic	25 (12)	13 (13)	12 (12)	
Breast	18 (9)	6 (6)	12 (12)	
Gynecologic	15 (7)	6 (6)	9 (9)	
Hematologic	6 (3)	1 (1)	5 (5)	
Head and neck	5 (3)	3 (3)	2 (2)	
Other	19 (10)	10 (10)	9 (9)	
Karnofsky Performance Status (mean [SD])	67 (10)	68 (9)	67 (10)	0.591^†^
Months since cancer diagnosis (median [interquartile range (IQR)]) (*n* = 197)	10 (3–25)	11 (3–23)	10 (3–33)	0.91^‡^
Months in palliative care (median [IQR]) (*n* = 197)	2 (0–7)	2 (0–5)	3 (0–10)	0.053^‡^

* chi-2; ^†^*t*-test; ^‡^Wilcoxon rank-sum test.

In bold: Statistically significant with p-value <0.05.

For time variables, including time since cancer diagnosis and time in PC, we performed a Shapiro–Wilk test for normality and in both cases the test rejected that the variables were normally distributed (*p* < 0.0001 in both cases). Therefore, to report these variables, we used median and IQRs. Median (IQRs) for time since cancer diagnosis and time in PC were 10 (3–25) months and 2 (0–7) months, respectively.

The mean scores for emotional distress, anxiety, and depression as measured with the HADS questionnaire are reported in Table 2. Forty-one percent of patients had clinically significant emotional distress (HADS total score). The proportion of patients with clinically significant anxiety and depression was 37% (HADS-A) and 45% (HADS-D), respectively.

**Table 2.** Patient report on received cancer treatments, perception of treatment goals, self-reported health status, and emotional distress

	Total sample	Accurate prognostic awareness	Inaccurate prognostic awareness	
	*N* = 201	*N* = 103	*N* = 98	
	*N* (%)	*N* (%)	*N* (%)	*p*-value*
Patient emotional distress: HADS total score (mean [SD])	13.9 (7.8)	15.2 (8.1)	12.5 (7.3)	**0.013**^†^
Patient anxiety: HADS-A score (mean [SD])	6.8 (4.1)	7.2 (4.3)	6.4 (3.9)	0.152^‡^
Patient depression: HADS-D score (mean [SD])	7.1 (4.4)	8 (4.7)	6.1 (4.1)	**0.003**^†^
In general, you would say your health is				0.282
Good	41 (20)	18 (17)	23 (23)	
Fair	88 (44)	43 (42)	45 (46)	
Bad	72 (36)	42 (41)	30 (31)	
Considering your disease, you could say You feel				**0.029**
Relatively healthy	49 (25)	25 (24)	24 (25)	
Relatively sick	99 (49)	43 (42)	56 (57)	
Very sick	53 (26)	35 (34)	18 (18)	
Cancer treatments received				
Surgery	115 (57)	58 (56)	57 (58)	0.791
Chemotherapy	88 (44)	43 (42)	45 (46)	0.551
Radiation therapy *N* = 200	57 (29)	27 (26)	30 (31)	0.46
Complementary/alternative medications *N* = 200	59 (30)	24 (23)	35 (36)	**0.048**
The goals of the treatments you are receiving are to get rid of the cancer. (*N* = 199)	94 (47)	26/102 (26)	68/97 (70)	**<0.001**
The goals of the treatments you are receiving are to help you live longer. (*N* = 199)	185 (93)	94/102 (92)	91/97 (94)	0.648
The goals of the treatments you are receiving are to help you improve your quality of life.	192 (96)	97 (94)	95 (97)	0.344

* chi-2; ^†^*t*-test.

In bold: Statistically significant with p-value <0.05.

Thirty-six percent of advanced cancer patients reported their health as bad, and 26% reported feeling very sick. Most patients reported that the goals of their therapies were to help them live longer (93%) and to improve their quality of life (96%). Ninety-four (47%) patients reported that treatments goals were to get rid of the cancer (Table 2).

In the univariate analysis, accurate PA was associated with marital status (*p* = 0.012), with patient with accurate PA having higher frequency of not having a partner than patients with inaccurate PA (46% versus 29%). We also found a trend for an association between PA and religion (*p* = 0.051). There were no associations between PA and other demographic characteristics. We did not find associations between PA with time since cancer diagnosis (*p* = 0.91) and with time in PC (*p* = 0.053). As these variables were not normally distributed, we transformed them using the logarithm of the observations. We did not find associations between PA with the log of time from cancer diagnosis or with the log of time in PC using a *t*-test (*p* = 0.62 and *p* = 0.25, respectively).

Accurate PA was also associated with increased emotional distress, using the HADS total score as a continuous (*p* = 0.013) or as a dichotomic variable (*p* = 0.047) and with increased depression, using the HADS-D total score as a continuous (*p* = 0.003) or dichotomic variable (*p* = 0.008), but not with anxiety (HADS-A total score: *p* = 0.152; HADS-A dichotomic variable; *p* = 0.454).

**Figure 1.**
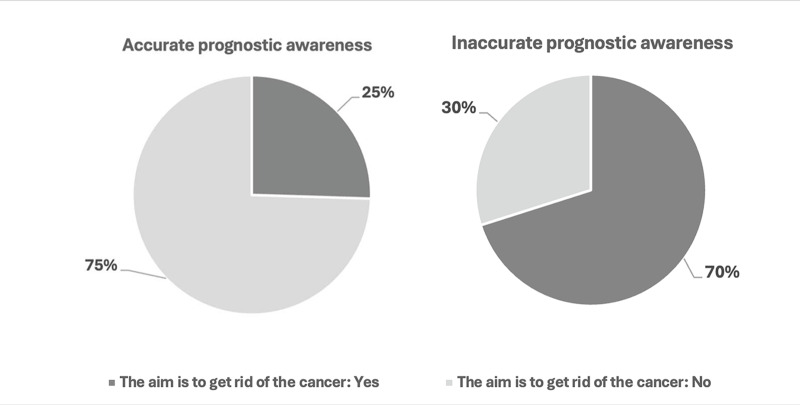
Patients Prognosis Awareness and Treatment Goals.

Patients with accurate PA reported more frequently being very sick compared to those with inaccurate PC (34% versus 18%, *p* = 0.029). Twenty-five percent of patients with accurate PA reported that the goals of their current treatments were to get rid of the cancer compared to 70% of those with inaccurate PA (*p* < 0.001) (Figure 1). Patients who believed that their cancer was not curable reported lower use of complementary/alternative treatments compared to those who believed that it was (23% versus 35%, *p* = 0.048).

In the multivariate analysis, we tested different models with PA as the main outcome. Most sociodemographic characteristics were unrelated to PA. Patients with a spouse or partner (OR; 95% CI; *p*-value/0.45; 0.23–0.86, 0.015) and those of Evangelical religion (0.43; 0.20–0.95; 0.038) were less likely to have an accurate PA (Table 3, model 1).

**Table 3.** Logistic regression models to explore the relationships between demographic characteristics with accurate prognostic awareness (PA) and the role of emotional distress

	Variables associated with accurate PA in Latino patients with advanced cancer and models including emotional distress as a predictor of PA.											
	*N* = 199											
	Model 1			Model 2			Model 3			Model 4		
	OR	95% CI	*p*	OR	95% CI	*p*	OR	95% CI	*p*	OR	95% CI	*p*
Female	0.77	0.41–1.47	0.433	0.90	0.47–1.74	0.754	0.86	0.45–1.65	0.649	0.86	0.45–1.66	0.660
Age	1.01	0.99–1.04	0.255	1.02	0.99–1.04	0.167	1.02	0.99–1.04	0.162	1.01	0.99–1.04	0.233
Education	0.78	0.40–1.53	0.471	0.77	0.38–1.54	0.345	0.76	0.38–1.52	0.444	0.78	0.39–1.55	0.472
With spouse/partner	0.45	0.23–0.86	**0.015**	0.45	0.23–0.87	**0.018**	0.42	0.22–0.82	**0.011**	0.48	0.25–0.93	**0.030**
Religion												
Catholic	-	-	-	-	-	-	-	-	-	-	-	-
Evangelical	0.43	0.20–0.95	**0.038**	0.45	0.20–1.01	0.054	0.44	0.20–0.99	**0.047**	0.45	0.20–1.00	0.051
Other	1.53	0.52–4.50	0.439	1.71	0.57–5.14	0.343	1.54	0.52–4.57	0.438	1.80	0.60–5.41	0.298
Atheist/agnostic	0.92	0.34–2.46	0.861	0.88	0.32–2.40	0.798	0.86	0.31–2.33	0.762	0.92	0.34–2.53	0.874
Religiosity	0.61	0.29–1.27	0.187	0.66	0.31–1.41	0.289	0.62	0.30–1.32	0.221	0.67	0.32–1.44	0.307
Spirituality	0.66	0.32–1.34	0.260	0.59	0.28–1.25	0.169	0.61	0.29–1.26	0.181	0.62	0.30–1.31	0.211
KPS	1.01	0.98–1.05	0.381	1.02	0.99–1.06	0.180	1.02	0.98–1.05	0.262	1.02	0.99–1.06	0.189
HADS score				1.07	1.02–1.13	**0.008**						
HADS-A score							1.10	0.99–1.20	0.054			
HADS-D score										1.12	1.03–1.21	**0.009**
Constant	1.26	0.07–23.6	0.877	0.21	0.01–5.41	0.349	0.42	0.02–9.66	0.584	0.30	0.01–6.95	0.454

OR = odds ratio; CI = confidence interval; KPS = Karnofsky Performance Status; HADS = Hospital Anxiety Depression Scale; HADS-A = HADS-anxiety; HADS-D = HADS-depression. Model 1: Original model including main demographic variables; Model 2: Original model with the addition of the HADS total score; Model 3: Original model with the addition of the HADS-Anxiety score; Model 4: Original model with the addition of the HADS-depression score.

In bold: Statistically significant with p-value <0.05.

We then included emotional distress, anxiety, and depression in the multivariate models to explore their associations with PA among patients with advanced cancer. We found that accurate PA was associated with increased emotional distress (1.07; 1.02–1.33; 0.008) and depression (1.12; 1.03–1.21; 0.009) but not anxiety (1.10; 0.99–1.20; 0.054) (Table 3, models 2–4). In all the multivariate models, having a spouse or partner remained significantly associated with PA (Table 3, models 2–4). Finally, in the multivariate models, we added the variable describing patient reports on treatment goals. In both models, patients with accurate PA were less likely to report that the goals of the treatments were to cure their cancer (OR; 95% CI; *p*-value/model 5: 0.13; 0.06; <0.001/model 6: 0.12; 0.06–0.25; <0.001). In these models, accurate PA remained associated with increased emotional distress (Table 4, model 5: 1.08; 1.02–1.14; 0.013) and depression (Table 4, model 6: 1.14; 1.03–1.25; 0.009). Also, in both models, having a spouse or partner but not religion was associated with a lower likelihood of having an accurate PA (Table 4, models 5 and 6). These models accounted for 24.3% and 24.6% of the total variance of the PA variable, respectively.

**Table 4.** Logistic regression models to explore the relationships between emotional distress and perceived goal of cancer treatments with perception of cancer curability

	Accurate prognostic awareness (PA) in Latino patients with advanced cancer					
	*N* = 199					
	Model 5			Model 6		
	OR	95% CI	*p*	OR	95% CI	*p*
Female	1.07	0.51–2.24	0.860	1.04	0.50–2.16	0.917
Age	1.02	0.99–1.05	0.123	1.02	0.99–1.05	0.185
Education	0.57	0.26–1.26	0.164	0.58	0.26–1.27	0.173
With spouse or partner	0.41	0.19–0.87	**0.021**	0.44	0.21–0.94	**0.033**
Religion						
Catholic	–	–	–	–	–	–
Evangelical	0.46	0.19–1.12	0.089	0.45	0.19–1.10	0.081
Other	1.48	0.44–5.00	0.524	1.55	0.46–5.27	0.482
Atheist/agnostic	0.93	0.30–2.90	0.897	0.93	0.30–2.93	0.905
Religiosity	0.81	0.35–1.86	0.614	0.82	0.36–1.89	0.648
Spirituality	0.59	0.26–1.33	0.203	0.62	0.27–1.40	0.245
KPS	1.02	0.98–1.06	0.264	1.02	0.98–1.06	0.267
HADS score	1.08	1.02–1.14	**0.013**			
HADS-D score				1.14	1.03–1.25	**0.009**
The goal of the treatment is to get rid of the cancer	0.13	0.06–0.26	**<0.001**	0.12	0.06–0.25	**<0.001**
Constant	0.69	0.02–26.2	0.843	0.97	0.03–32.5	0.986

OR = odds ratio; CI = confidence interval; KPS = Karnofsky Performance Status; HADS = Hospital Anxiety Depression Scale; HADS-A = HADS-anxiety; HADS-D = HADS-depression. Model 5: Original model with the addition of total HADS score and the variable about goals of treatment. Model 6: Original model with the addition of total HADS-depression score and the variable about goals of treatment.

In bold: Statistically significant with p-value <0.05.

## Discussion

This study reveals that half of a population of advanced cancer patients in a Latino community reported having an accurate PA, like what has been reported elsewhere (Chen et al. 2017). This study also shows an association between emotional distress with PA in this population. In fact, with each unit increase in the HADS and HADS-D scored, the likelihood of a perception of accurate PA increased by an adjusted odd ratio of 1.05 and 1.11, respectively. We also found that patients with a partner and those who thought that the goals of the treatments were to get rid of the cancer were less likely to have accurate PA.

Although little more than 50% of advanced cancer patients of this Latino community reported having accurate PA, a similar percentage still believed that their cancer was curable, when, in fact, it was not. In this population, patients who had a spouse or partner were more likely to believe that their cancer was curable. These findings are consistent with research reporting that patients with higher social contact, such as having a partner, make lacking PA more likely (Chochinov et al. 2000). Latino cultures are characterized by intense social relationships and increased family and community involvement in patient care (Torres Blasco et al. 2023). Two publications describing advanced cancer patient preferences for decision-making in Chile, one in a public and the other in a private institution, reported that a high proportion of patients preferred shared or passive decision-making styles, meaning that the involvement of family members or physicians was frequently needed (Yennurajalingam et al. 2013; Yennurajalingam et al. 2018a). Consequently, having strong interpersonal bonds can make the process of incorporating prognostic information more challenging for patients, as this might be a family or a community process rather than an individualistic one in this culture.

A relevant finding in this study is the high proportion of patients reporting clinically significant emotional distress and depression and its association with accurate PA, which is similar to previous findings in some studies around the globe (Chan 2011; El‐Jawahri et al. 2014; Kang et al. 2020; Nipp et al. 2017; Ozdemir et al. 2022). From these results, it is unclear whether extensively promoting PA across Latin American cultures could benefit patients in the same way it has been proposed in other contexts. Several hypotheses can be proposed to explain this association. First, reports show that one key aspect that affects PA and emotional well-being is the way prognostic information is disclosed to patients and family members. Providing prognostic information tailored to what patients need, with a patient-centered perspective may improve patient understanding and acceptance of their status and could have positive impacts in patients’ emotional well-being. The lack of training in PC and in communication skills in Chile and in Latin America it an important gap that need to be urgently addressed. A second hypothesis that could be proposed is that PA is a longitudinal process through which patients understand and are able to gain acceptance. In this cross-sectional study, we assessed PA at one time point and were unable to assess whether this negative association between PA and emotional well-being persisted or changed over time, as reported in other studies (George et al. 2020). A third proposed hypothesis could be the role of culture influencing this association. We could hypothesize that hope could be an attribute that protects patients from emotional distress or depression associated with PA. This could be the case in cultures with strong religious backgrounds such as Latin America where spirituality and religiosity are frequent, intense, and rarely addressed in this population (Delgado-Guay et al. 2021).Therefore, identifying cultural characteristics playing a role in modulating emotional distress when providing prognostic information, such as hope, could help clinicians develop culturally appropriate interventions to support patients from different cultural backgrounds (Butow et al. 2020; Hui et al. 2021; Walczak et al. 2013). A final hypothesis could be that advanced cancer patients with higher emotional distress were motivated to seek for more prognostic information, and therefore were able to have an increased PA.

Another relevant finding in this study was that we found a strong association between patients reporting that their cancer was curable (lack of PA) and patients reporting that the goal of the treatment interventions were to get rid of the disease. Although we describe just an association between variables, it is possible to propose that one of the reasons for the high frequency of inaccurate PA is the lack of understanding of this population about the meaning of prognosis or curability due to the low literacy reported. Prognosis and curability are complex concepts which require understanding abstract ideas that might be difficult to comprehend for people with low education. Another possible explanation for the association between lack of PA with believing that the treatment goals were to get rid of the disease is that clinicians have provided information not clear enough to this population. In Chile, like in most Latin American countries, there are few oncologists available meaning that they are usually overloaded with clinical work and have little time to spend with patients to explain their conditions, prognosis and to help them in the decision-making process (Ministerio de Salud 2018). The scarcity of communication training in oncology could also influence the way this complex information is delivered to patients (Gilligan et al. 2017).

This study is not free of limitations. First, it was performed at a single site in a single country affecting the results’ generalizability. Therefore, these results do not represent the reality of the whole country or region. Nevertheless, this is the first study describing the reality of PA in a Latino population of advanced cancer patients, which could contribute to promoting the discussion of this topic in the region. Second, the design of this study was cross-sectional, allowing us to report only associations between variables and not to propose a causal relationship between them. Third, we used a single yes/no question to assess patients’ PA, affecting the reliability of our findings. Recent literature describes that many strategies are used to assess PA, including single or multiple items, so using more than one instrument to assess the phenomenon could have strengthened our findings. We did include a different set of questions to assess patient perception of treatment goals, and one of them – asking whether the goal of the treatments was to get rid of their cancer – has been used in some studies to assess PA. The fact that this variable was significantly associated with our definition of PA, strengthens our results, and partially overcomes this limitation. Fourth, our final model accounts for a limited proportion of the observed PA, suggesting that other variables could influence this outcome. Finally, we did not assess other relevant covariates, such as hope or patient informational needs, that could have contributed to better understand this complex phenomenon. This information is relevant to provide better guidance for clinicians who frequently deliver prognostic information.

In conclusion, this study reveals that half of a population of advanced cancer patients in a Latino community reported having an accurate PA which was associated with increased emotional distress and depression. Overall, these results have implications for advanced cancer practitioners in Chile and Latin America. Disclosing prognostic information may negatively impact patients’ emotional well-being, and therefore, strategies to support and accompany patients emotionally throughout the process will be relevant. Improving communication training for oncologists could improve the way the prognostic information is disclosed. Interventions such as screening for emotional distress in follow-up assessments should be considered as a regular practice after prognostic conversations occur.

## Data Availability

Data used in this study are part of Proyecto Fondecyt de Iniciación No. 11130533 “Measuring Quality of Dying and Death in Patients With Advanced Cancer – Association Between After Death Caregiver Reported Quality of Dying and Patient Reported End-of-Life Care Outcomes.” The data that support the findings of this study are available on request from the corresponding author. The data are not publicly available due to privacy or ethical restrictions.
